# The clinical impact of self‐reported symptoms of chronic rhinosinusitis in people with bronchiectasis

**DOI:** 10.1002/iid3.547

**Published:** 2021-10-14

**Authors:** Annemarie L. Lee, Caroline H. H. Nicolson, Janet Bondarenko, Brenda M. Button, Samantha Ellis, Robert G. Stirling, Mark Hew

**Affiliations:** ^1^ Department of Physiotherapy, Faculty of Medicine, Nursing and Health Sciences, School of Primary and Allied Health Care Monash University Frankston Australia; ^2^ Institute for Breathing and Sleep Austin Health Heidelberg Australia; ^3^ Centre for Allied Health Research and Education Cabrini Health Malvern Australia; ^4^ Department of Allergy, Asthma and Clinical Immunology Alfred Health Melbourne Australia; ^5^ Department of Physiotherapy Alfred Health Melbourne Australia; ^6^ Department of Medicine, Faculty of Medicine, Nursing and Health Sciences Monash University Frankston Australia; ^7^ Department of Radiology Alfred Health Melbourne Australia; ^8^ Sub‐Faculty of Translational Medicine and Public Health Monash University Frankston Australia

**Keywords:** bronchiectasis, pneumology, quality of life, rhinosinusitis

## Abstract

**Background:**

Chronic rhinosinusitis affects 62% of adults with bronchiectasis and is linked to greater bronchiectasis severity. However, the impact of symptoms of chronic rhinosinusitis on disease‐specific and cough‐related quality of life is unknown.

**Methods:**

In this cross‐sectional study, adults with stable bronchiectasis and chronic rhinosinusitis symptoms completed the sinonasal outcome test‐22 (SNOT‐22), quality of life–bronchiectasis questionnaire, and Leicester cough questionnaire. Bronchiectasis severity was assessed using the bronchiectasis severity index (BSI) and chest high‐resolution computed tomography (HRCT).

**Results:**

Sixty participants with bronchiectasis (mean [SD] forced expiratory volume in 1 s of 73.2 [25.5] %predicted) were included. Greater severity of chronic rhinosinusitis symptoms (based on SNOT‐22) was moderately associated with impaired cough‐related quality of life (according to the Leicester cough questionnaire; all *r* > −.60) and impaired bronchiectasis‐specific quality of life (based on the quality of life–bronchiectasis questionnaire), with impaired physical function (*r* = −.518), less vitality (*r* = −.631), reduced social function (*r* = −.546), greater treatment burden (*r* = −.411), and increased severity of respiratory symptoms (*r* = −.534). Chronic rhinosinusitis symptoms were unrelated to disease severity according to the BSI (*r* = .135) and HRCT scoring (all *r* < .200). The severity of chronic rhinosinusitis symptoms was not affected by sputum color (*p* = .417) or the presence of *Pseudomonas aeruginosa* colonization (*p* = .73).

**Conclusions:**

In adults with bronchiectasis, chronic rhinosinusitis has a consistent and negative impact on both cough‐related and bronchiectasis‐specific quality of life.

## 1 INTRODUCTION

Bronchiectasis is a chronic respiratory disease characterized by abnormal destruction and dilatation of the bronchi and bronchioles,^1^ which develops secondary to airway inflammation, infection, and mucociliary dysfunction. Common symptoms include chronic sputum production and recurrent respiratory infections, which contribute to progressive disease and reduced health‐related quality of life.^2^, ^3^ While lower airway manifestations of bronchiectasis are hallmark features, upper airways symptoms, including chronic rhinosinusitis have been recognized as part of the clinical presentation with specific aetiologies of bronchiectasis.^4^, ^5^, ^6^

Chronic rhinosinusitis is an inflammatory condition of the nasal passages and paranasal sinuses lasting for 12 weeks or more, with the key feature of inflammation of the nasal cavity and paranasal sinuses.^7^ Cardinal symptoms for diagnosis of chronic rhinosinusitis include anterior/posterior mucopurulent drainage, nasal obstruction, face pain/pressure/fullness, and hyposmia, together with endoscopic or radiological evidence of mucosal inflammation.^8^ The prevalence of chronic rhinosinusitis in people with bronchiectasis is 62%.^9^ Linkage between upper and lower airway disease in bronchiectasis is suggested by more extensive bronchiectasis (greater number of lobes affected)^4^ and poorer lung function as reflected by spirometry^10^ in those with coexistent rhinosinusitis. While this provides an indication of the negative impact of upper airway symptoms on disease severity, the relationship between chronic rhinosinusitis symptoms and detailed measurement of bronchiectasis severity and extent has not been explored.

Chronic rhinosinusitis has a negative impact on the physical, psychological and social aspects of patients’ lives, with reports of greater pain and poorer social functioning.^11^ In patients with bronchiectasis, the presence of chronic rhinosinusitis confers a poorer overall health‐related quality of life, as reflected by disease‐specific and generic measures.^12^, ^13^ However, the relationship between chronic rhinosinusitis symptoms and cough‐related quality of life^14^ in people with bronchiectasis has not been evaluated. Gaining an understanding of these relationships will provide further insight into the clinical value of assessing chronic rhinosinusitis symptoms in bronchiectasis.

This study aimed to establish the relationship between chronic rhinosinusitis symptoms, bronchiectasis severity, and disease‐specific and cough‐related quality of life in adults with bronchiectasis and symptoms of chronic rhinosinusitis.

## 2 METHODOLOGY

### 2.1 Study design and setting

This was a prospective cross‐sectional study undertaken within the bronchiectasis outpatient clinic at Alfred Health between October 2016 and July 2018. This study was conducted according to the guidelines of the Declaration of Helsinki and approved by the Institutional Human Research Ethics committee of Alfred Health (protocol number 123/17, March 22, 2017). All participants provided written informed consent before participation.

### 2.2 Participants

Patients were invited to participate if they were aged over 18 years, had a primary clinical respiratory diagnosis of bronchiectasis, confirmed on high‐resolution computed tomography (HRCT) based on published criteria,^15^ and presented with chronic rhinosinusitis symptoms. Participants were clinically stable, and concomitant respiratory conditions of chronic obstructive pulmonary disease (COPD) or asthma were noted. Confirmation of chronic rhinosinusitis symptoms included two or more of the following signs or symptoms for 12 weeks: mucopurulent discharge, nasal obstruction, facial pain/pressure, and decreased sense of smell.^16^, ^17^ Individuals with a diagnosis of cystic fibrosis (sweat test or genetic testing); bronchiectasis diagnosed by symptoms alone unsupported by computed tomography (CT); or hospitalization or treatment for an acute exacerbation within the previous four weeks were all excluded.

The underlying etiology of bronchiectasis was extracted from the participant's medical records. Age, gender, and body mass index were recorded at the time of measurement. Sputum color was assessed using a reliable and validated color chart representing three typical gradations of color: mucoid, mucopurulent, and purulent.^18^ Color selection was recorded for the day of assessment. Treatments for chronic rhinosinusitis symptoms were recorded, as documented within the participants’ medical records and directly from the participant.

### 2.3 HRCT—chest and paranasal

HRCT scoring of the chest and paranasal sinus scans were completed by an experienced radiologist, who was blinded to the presence of chronic rhinosinusitis symptoms in participants. On the chest scan, the extent of bronchiectasis was scored according to the following criteria: number of bronchopulmonary segments, severity, the extent of peribronchiolar thickening, generations of bronchial divisions involved, sacculations or abscesses, number of bullae, and extent of emphysema.^15^ A subgroup of patients with paranasal sinus scans had their images graded according to the Lund–Mackay scoring system of 0–24.^19^ Scans were undertaken as part of routine clinical practice within the last 5 years.

Participants completed the following measurements during a single outpatient clinic visit.

### 2.4 Bronchiectasis severity index

This tool produces a score of severity based on age, body mass index, lung function, previous hospital admission for severe exacerbation, number of exacerbations, medical research council breathlessness scale, radiological information, and colonization with *Pseudomonas aeruginosa* or other bacteria.^20^ Age, body mass index, and rating of dyspnea were obtained directly from the participant. Spirometry measures completed as part of routine clinical practice, according to American Thoracic Society guidelines^21^ in the 6 months before study recruitment were used. At the time of spirometry testing, all participants were classed as clinically stable. Reports of the previous hospitalization for exacerbations and the number of exacerbations for the bronchiectasis severity index (BSI) were obtained from participant reports and confirmed from the medical record. Radiological information was obtained from the participants’ chest scans. The most recent microbiological, culture, and sensitivity were assessed to identify colonial morphology of sputum samples as noted from the participant's clinical records.

### 2.5 Sino‐nasal outcome test‐22 (to assess the severity of chronic rhinosinusitis symptoms)

This questionnaire includes 22 questions related to the symptom and impact of sinusitis, with each item scored using a 0–5 point Likert scale. There are four subscales (rhinological symptoms [need to blow nose, nasal blockage, sneezing, runny nose, cough, thick nasal discharge, and ear fullness], ear/facial symptoms [dizziness, ear pain, facial pain/pressure, and decreased sense of smell/taste], psychological function [fatigue, reduced productivity, reduced concentration, frustrated/restless/irritable, sad, and embarrassed], and sleep function [difficulty falling asleep, waking up at night, lack of a good night's sleep] and a total score (ranging from 0 to 110). Higher scores indicate a greater rhinosinusitis‐related health burden,^22^, ^23^ with classifications of mild disease based on scores of 8–17, moderate 22.5–48, and severe 54–83.^24^

### 2.6 Quality of life questionnaire–bronchiectasis (to assess the bronchiectasis‐specific quality of life)

This questionnaire consists of 37 items with 8 scales, each scale scored from 0 to 100, with higher scores indicating better function and quality of life. Validity and reliability have been established for this tool.^25^

### 2.7 Leicester cough questionnaire (to assess the cough‐related quality of life)

This is validated in people with bronchiectasis^14^ and consists of 19 questions with three subscales (physical, social, and psychological, ranging from 1 to 7) and a total score (ranging from 3 to 21), with lower scores indicating greater impairment.

### 2.8 Statistical analysis

An estimated sample size of 60 participants was calculated as necessary to establish a significant correlation of *r* > .80 between the presence of chronic rhinosinusitis and quality of life.^26^ Data were analyzed using SPSS for Windows (SPSS 25.0). Data were presented as mean (standard deviation [SD]) for normally distributed data or median (interquartile range) for non‐normally distributed data. Pearson's correlation coefficient was used to examine the relationships between severity of chronic rhinosinusitis symptoms on sinonasal outcome test‐22 (SNOT‐22), bronchiectasis‐specific, and cough‐related quality of life, and bronchiectasis severity on HRCT and BSI. The strength of association was classed as weak (*r* = .1–.29), moderate (*r* = .3–.49), or strong (*r* = .5–1.0).^27^ Due to multiple comparisons, a Bonferroni correction was applied and the significance level was set at *p* < .003. Difference between classifications of SNOT‐22 total scores (mild, moderate, and severe),^24^ sputum color (mucoid, mucopurulent, and purulent), presence or absence of *Pseudomonas aeruginosa* colonization, and classifications of bronchiectasis severity (based on BSI) were analyzed using Chi square. Statistical significance was set at an *α* < .05 unless otherwise stated.

## 3 RESULTS

### 3.1 Participant characteristics

**FIGURE 1:**
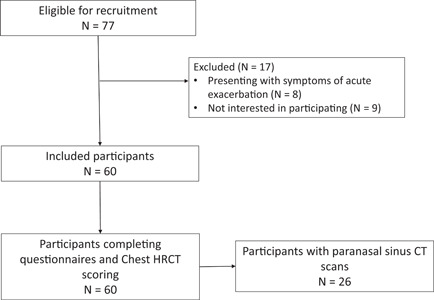
Flow of participants. CT, computed tomography; HRCT, high‐resolution computed tomography

A total of 60 participants were recruited for this study, with Figure 1 depicting the recruitment flow. Of these 65% were female, with a mean (SD) age of 59 (18) years, and a mean forced expiratory volume in 1 s of 73.2 (25.5) %predicted (Table 1). Common aetiologies were postinfection (38%) and idiopathic (20%), with 17% unclassified. The most common organism identified in the participant's sputum was *Pseudomonas aeruginosa* (32%), followed by *Staphylococcus aureus* (7%) and *Haemophilus influenza* (7%), with the majority (80%) of participants reporting mucoid sputum on the day of assessment. The mean severity of bronchiectasis according to the BSI was moderate. A mix of treatment options was currently being applied by participants for the management of chronic rhinosinusitis symptoms (Table 1). Seven participants had previously had endoscopic sinus surgery.

**TABLE 1:** Participant demographics

Demographic	Mean (SD) or *N* (%)
Female/male	39 (65%)/21 (35%)
Age, y	58 (18)
BMI (kg/m^2^)	24.7 (5.9)
FEV_1_ %predicted	73.2 (25.5)
FVC %predicted	82.8 (19.9)
FEV_1_/FVC	66.1 (18.9)
BSI (points)	6.5 (4.3)
Etiology of bronchiectasis	
Postinfection	23 (38%)
Immunodeficiency	8 (13%)
Primary ciliary dyskinesia	7 (12%)
Idiopathic	12 (20%)
Not classified	10 (17%)
Microbes	
*Pseudomonas aeruginosa*	19 (32%)
*Staphylococcus aureus*	4 (7%)
*Haemophilus influenzae*	4 (7%)
*Aspergillus*	2 (3%)
*Streptococcus*	2 (3%)
*Mycobacterium avium* complex	2 (3%)
Sputum color	
Mucoid	48 (80%)
Mucopurulent	6 (10%)
Purulent	4 (10%)
Chronic rhinosinusitis treatment	
Nasal saline irrigation	28 (47%)
Intranasal corticosteroid spray	13 (22%)

Abbreviations: BMI, body mass index; BSI, bronchiectasis severity index; FEV_1_, forced expiratory volume in 1 s; FVC, forced vital capacity; *N*, number; SD, standard deviation.

Scores for each of the domains of the quality of life–bronchiectasis questionnaire are outlined in Table 2. The degree of impairment in cough‐related quality of life according to the Leicester cough questionnaire was moderate^28^ (Table 2). The severity of rhinosinusitis according to the sinonasal outcome test was moderate for the total scores. The extent and severity of bronchiectasis according to HRCT scoring and severity of paranasal sinus disease are outlined in Table 2.

**TABLE 2:** Quality of life, sinonasal questionnaire scores, and computed tomography scoring

Variable	Mean (SD) or *N* (%)
Quality of life–bronchiectasis	
Physical function	63.9 (30.5)
Role functioning	64.3 (22.6)
Vitality	50.4 (26.9)
Emotional function	77.6 (21.5)
Social functioning	56.4 (24.5)
Treatment burden	54.9 (27.8)
Health perceptions	49.3 (23.6)
Respiratory symptoms	60.6 (19.8)
Leicester cough questionnaire	
Physical score	4.9 (1.3)
Psychological score	5.2 (1.5)
Social score	5.3 (1.3)
Total score	15.4 (3.9)
Sinonasal outcome test	
Rhinological symptoms	14.5 (8.2)
Ear/facial symptoms	5.4 (5.1)
Sleep function	8.3 (7)
Psychological function	7.9 (7.2)
Total score	36.5 (23.6)
Computed tomography scoring	
Chest	
Extent of bronchiectasis	1–5 segments (12 [20%]); 6–9 segments (7 [12%]); >9 (15 [25%])
Severity of bronchiectasis	Mild: 14 (23%); moderate: 13 (22%); severe: 7 (12%)
Peribronchial thickness	Mild: 32 (53%); moderate: 2 (3%); severe: 1 (2%)
Sacculations	Absent: 34 (57%); 1–5: 1 (2%)
Generations	4th gen: 5 (8%); 5th gen: 10 (17%); 6th gen: 19 (32%)
Bullae	Absent: 34 (57%); unilateral: 1 (2%)
Emphysema	Absent: 33 (55%); >5: 2 (3%)
Collapse and consolidation	Absent: 15 (25%); subsegmental: 2 (3%); segmental: 18 (30%)
Extent of mucus plugging	Absent: 16 (27%); 1–5: 16 (27%); 6–9: 1 (2%); >9: 2 (3%)
Paranasal sinuses^a^	
Right maxillary sinus	Normal: 6 (10%); partial opacity: 16 (27%); total opacity: 4 (7%)
Left maxillary sinus	Normal: 9 (15%); partial opacity: 15 (25%); total opacity: 2 (3%)
Right anterior ethmoid	Normal: 11 (18%); partial opacity: 11 (18%); total opacity: 4 (7%)
Left anterior ethmoid	Normal: 12 (20%); partial opacity: 9 (15%); total opacity: 5 (8%)
Right sphenoid	Normal: 9 (15%); partial opacity: 14 (23%); total opacity: 3 (5%)
Left sphenoid	Normal: 11 (18%); partial opacity: 13 (22%); total opacity: 2 (3%)
Right frontal sinus	Normal: 18 (30%); partial opacity: 4 (7%); total opacity: 4 (7%)
Left frontal sinus	Normal: 16 (27%); partial opacity: 5 (8%); total opacity: 5 (8%)
Ostiomeatal complex	No obstruction: 16 (27%); obstruction: 10 (17%)
Absent frontal sinus	Absent: 24 (40%); present: 2 (3%)
Concha bullosa	Absent: 22 (37%); present: 4 (7%)
Paradoxical middle turbinates	Absent: 20 (33%); present: 6 (10%)
Haller cells	Absent: 25 (42%); present: 1 (2%)
Everted uncinated process	Absent: 23 (38%); present: 3 (5%)
Agger nasi pneumatisation	Absent: 13 (22%); present 13 (22%)

^a^ Only *n* = 26 had paranasal sinus computed tomography scans.

### 3.2 Relationships between chronic rhinosinusitis, quality of life, and CT severity

Greater severity of chronic rhinosinusitis symptoms was moderately associated with impaired cough‐related quality of life, reduced physical and social function, less vitality, greater treatment burden, and increased severity of respiratory symptoms, and weakly associated with a poorer perception of health and impaired emotional function (Table 3). Chronic rhinosinusitis symptoms were poorly related to the BSI (*r* = .135) and bronchiectasis HRCT scoring (all *r* < .200). There was no difference in classification of chronic rhinosinusitis symptoms according to sputum color (*p* = .417) or the presence or absence of *Pseudomonas aeruginosa* colonization (*p* = .730).

**TABLE 3:** Relationship between sinus symptoms and quality of life measures

Cough‐related quality of life				Bronchiectasis‐specific quality of life							
	Physical	Psychological	Social	Physical	Role	Vitality	Emotional function	Social	Treatment burden	Health perception	Respiratory symptoms
SNOT‐22 domains and total score											
Rhino	−.506	−.399	−.434	−.431	−.366	−.472	−.147	−.326	−.247	−.265	−.484
	*p* < .001	*p* = .002	*p* = .001	*p* = .001	*p* = .004	*p* < .01	*p* = .26	*p* = .01	*p* = .08	*p* = .04	*p* < .01
Ear	−.549	−.563	−.612	−.440	−.446	−.555	−.372	−.525	−.349	−.410	−.424
	*p* < .01	*p* < .01	*p* < .01	*p* < .01	*p* < .01	*p* < .01	*p* = .00	*p* < .01	*p* = .01	*p* = .002	*p* = .001
Sleep	−.640	−.585	−.552	−.429	−.332	−.648	−.111	−.543	−.471	−.401	−.471
	*p* < .01	*p* < .01	*p* < .01	*p* = .001	*p* = .01	*p* < .01	*p* = .40	*p* < .01	*p* < .01	*p* = .002	*p* < .01
Psychological	−.610	−.609	−.632	−.450	−.358	−.511	−.299	−.526	−.362	−.285	−.446
	*p* < .01	*p* < .01	*p* < .01	*p* < .01	*p* = .005	*p* < .01	*p* = .02	*p* < .01	*p* = .01	*p* = .03	*p* < .01
Total	−.673	−.625	−.641	−.518	−.430	−.631	−.258	−.546	−.411	−.379	−.534
	*p* < .01	*p* < .01	*p* < .01	*p* < .01	*p* = .01	*p* < .01	*p* = .05	*p* < .01	*p* = .00	*p* = .003	*p* < .01

*Note*: Data are Pearson's *r* correlation coefficient, due to multiple comparisons, significance levels were set at *p* < .003.

Abbreviation: SNOT‐22, sinonasal outcome test‐22.

## 4 DISCUSSION

In people with bronchiectasis, the presence of chronic rhinosinusitis symptoms is associated with impaired cough‐related and disease‐specific quality of life, highlighting the impact of the sinonasal disease. The presence of chronic rhinosinusitis symptoms is not associated with bronchiectasis disease severity according to the BSI or HRCT scoring.

The negative impact of chronic rhinosinusitis symptoms on disease‐specific quality of life found in this study builds on earlier reports of impaired physical and mental health in people with bronchiectasis and chronic rhinosinusitis.^12^, ^13^, ^29^ However, unlike generic tools, the quality of life–bronchiectasis tool was specifically developed for people with bronchiectasis to assess symptoms, function, and health‐related quality of life.^25^ The strong correlation between chronic rhinosinusitis symptoms and physical symptoms, vitality, social function, and respiratory symptoms highlights the physical and social burden of these symptoms. A similar relationship has been demonstrated in individuals with cystic fibrosis and COPD,^30^, ^31^, ^32^ with a poorer perception of respiratory health in those with chronic sinus disease.^30^

The moderate‐to‐strong associations between cough‐related quality of life and chronic rhinosinusitis symptoms further highlight the negative impact of these symptoms, even in the absence of consistent paranasal CT scans or nasal endoscopy to establish the severity of disease on an important patient‐reported outcome. Recent studies highlighted the importance of cough as a determinant of quality of life in bronchiectasis,^32^ as it is recognized as a dominant symptom of bronchiectasis linked to social embarrassment and sleep disturbance.^33^, ^34^ In people with cystic fibrosis, nasal obstruction, face pain or pressure, headaches, mucopurulent discharge, and postnasal drip have been associated with poor sleep and activity intolerance.^35^, ^36^ Similar associations are present in this study of adults with bronchiectasis, based on the physical, psychological, and social burden of coughing. This is evident despite a proportion of participants currently prescribed treatment to manage their chronic rhinosinusitis symptoms or having undertaken previous surgery. This highlights the relevance of including both disease‐specific and symptom‐specific tools to assess the impact of chronic rhinosinusitis symptoms in bronchiectasis.

It has been suggested that the upper and lower airways in bronchiectasis are linked from a radiological view.^37^ The lack of association between bronchiectasis severity and chronic rhinosinusitis symptoms is similar to some earlier studies,^38^, ^39^ but not others.^10^, ^29^ This may be influenced by current or previous treatment to manage chronic rhinosinusitis symptoms in some participants. Without endoscopy or paranasal CTs for all participants, the severity of chronic rhinosinusitis is unclear and interpretation of the relationship between the upper and lower airways is limited. In addition, this discrepancy may reflect the variety of approaches to HRCT scoring undertaken to assess the severity of bronchiectasis. Some tools are designed to examine in detail the severity and extent of bronchiectasis,^15^, ^40^, ^41^ as was applied in the current study, while others are principally designed for diagnostic purposes.^4^, ^42^ The absence of a relationship between BSI and chronic rhinosinusitis symptoms has been previously documented in those with bronchiectasis and COPD.^38^ In people with COPD, no significant relationship between measures of spirometry and nasal symptoms has been demonstrated.^31^, ^43^ This may suggest that chronic rhinosinusitis symptoms have no impact on respiratory function or severity of disease in a group of individuals with bronchiectasis, some of whom are receiving or have received treatment for these symptoms. It also suggests that alternative measures of disease severity, in the form of exacerbations, may be better suited to accurately describe this relationship. Indeed, patients with both bronchiectasis and chronic rhinosinusitis symptoms have been observed to be at higher risk of acute exacerbations.^38^ Further exploration of the relationship between upper airway symptoms and various markers of acute exacerbations, including time to first exacerbation, frequency, and severity in those with bronchiectasis, is clearly required.

A higher prevalence of *Pseudomonas aeruginosa* has been isolated in people with bronchiectasis with a diagnosis of chronic rhinosinusitis, based on EP3OS criteria.^38^ Conversely, other studies noted no difference in sputum colonization for *Pseudomonas aeruginosa* in those with or without upper airway symptoms.^12^, ^39^ The small proportion of participants with *Pseudomonas aeruginosa* colonization (32%) limits any interpretation of this relationship. Similarly, with a dominance of mucoid sputum reported by participants in the current study, a larger number of participants would be required to accurately interpret the relationship between the severity of chronic rhinosinusitis symptoms and sputum color.

The quality of life of people with bronchiectasis is negatively affected by respiratory symptoms and treatment burden.^44^ Gaining some insight into the possible contribution of upper airway symptoms to health‐related quality of life highlights the value of assessment of chronic rhinosinusitis symptoms as part of clinical practice. This lends further support to studies examining the management of chronic rhinosinusitis symptoms on this important patient‐reported outcome.

We acknowledge several limitations to this study. Participants were recruited from a single tertiary center only; the extent to which these findings apply for those with bronchiectasis and symptoms of chronic rhinosinusitis in other centers is unclear. Some participants were recipients of current or past treatment for chronic rhinosinusitis symptoms, which is likely to influence the reporting of symptom severity. Participants were recruited based on their report of chronic rhinosinusitis symptoms; nasal endoscopy to enable further examination or confirm diagnosis was not included. For these reasons, the generalizability of the findings should be interpreted with caution. We included individuals with a primary diagnosis of bronchiectasis, including those with primary ciliary dyskinesia.^45^ This, together with comorbid respiratory conditions, including asthma, COPD, allergens or gastro‐esophageal reflux disease,^46^, ^47^ smoking history,^48^ and frequency of acute exacerbations of bronchiectasis,^9^ are all factors which are associated with chronic rhinosinusitis and may have contributed to the symptoms reported on the SNOT‐22 questionnaire. In the absence of collating this information, the extent to which the underlying cause of bronchiectasis and comorbid conditions contributed to symptoms of chronic rhinosinusitis or quality of life scores is not clear. Paranasal sinus CT scans were only available for 43% of participants in this study. This limited full exploration of the radiological extent of chronic rhinosinusitis and its impact on quality of life.

In conclusion, individuals with bronchiectasis and chronic rhinosinusitis symptoms have impaired disease‐specific quality of life and cough‐related quality of life. Future studies may further explore the impact of chronic rhinosinusitis symptoms and markers of acute exacerbations of bronchiectasis.

## CONFLICT OF INTERESTS

The authors declare that there are no conflict of interests.

## AUTHOR CONTRIBUTIONS

Annemarie L. Lee, Caroline H. H. Nicolson, Janet Bondarenko, and Mark Hew conceived the study design. Annemarie L. Lee, Caroline H. H. Nicolson, Janet Bondarenko, and Samantha Ellis collated the data. Annemarie L. Lee undertook the data analysis and drafted the manuscript. All authors contributed to the interpretation and the final manuscript.

## Data Availability

The data that support the findings of this study are available from the corresponding author upon reasonable request.
